# A flexible and visually meaningful multi-image compression, encryption and hiding scheme based on 2D compressive sensing

**DOI:** 10.1016/j.heliyon.2023.e14072

**Published:** 2023-02-27

**Authors:** Dongming Huo, Zhilong Zhu, Xin Zhou, Lisheng Wei, Xing Bai, Yanzhi Bai, Chao Han

**Affiliations:** a Key Laboratory of Advanced Perception and Intelligent Control of High-End Equipment, Ministry of Education, College of Electrical Engineering, Anhui Polytechnic University, Wuhu 241000, China; b Department of Opto-Electronics Science and Technology, Sichuan University, Chengdu 610065, China; c College of Science, Henan University of Technology, Zhengzhou 450001, China

**Keywords:** Multi-image encryption, Compressive sensing, Visually meaningful cipher image

## Abstract

**Background:** Encrypting plain images into noise-like cipher images is a common method in image encryption. However, when noise-like images appear in public networks, they are more likely to attract attention and suffer more cryptanalysis. To solve this problem, researchers propose the concept of visually meaningful image encryption scheme, which encrypts a plain image into a visually meaningful cipher image. **Objective:** In order to realize the visual security of cipher image and increase information capacity, this paper proposes a flexible visually secure multi-image compression, encryption and hiding scheme based on two-dimensional compressive sensing (2DCS), which can flexibly complete the compression and encryption of multiple plain images without increasing the amount of ciphertext data. **Methods:** The scheme is divided into encryption process and embedding process. In the encryption process, the plain image is randomly scrambled and non-linear gray value transformed to obtain a pre-encrypted integer matrix, then 2DCS is used to compress the pre-encrypted integer matrix to get the secret image. Repeat this process for multiple plain images to obtain multiple secret images. In the embedding process, integer wavelet transform and bit-plane decomposition are used to embed multiple secret images into the quantized coefficient matrix of host image to get the modified coefficient matrix, and then the inverse integer wavelet transform is used to transform the modified coefficient matrix into spatial space to get the visually meaningful cipher image. **Result:** The simulation experiment verifies the feasibility and effectiveness of the visually meaningful multi-image encryption scheme, and users can choose to improve the system's encryption capacity or cipher image's visual security according to their own needs.

## 1 Introduction

### 1.1 Background

Today, with the rapid development of information technology, more and more people transmit images and other information through the network and store them on the server. When transmitting such information, it is sometimes hoped that no one but the recipient can see the transmitted information. Therefore, how to solve the security problem in the process of information transmission has become an important challenge. Image encryption is one of the important means to ensure the security and confidentiality of image information.

**Figure 1:**
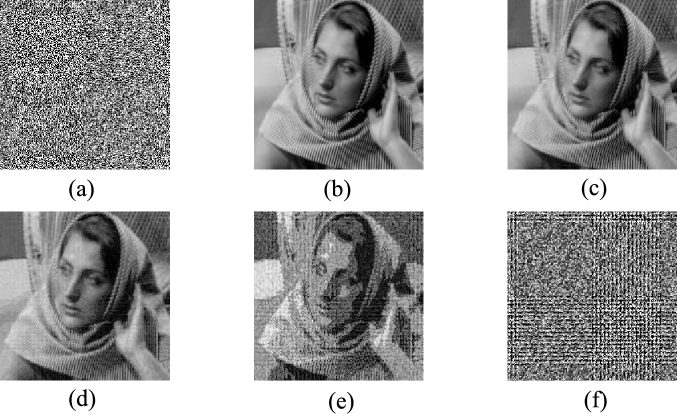
(a) Noise-like cipher image; (b) host image; (c)–(f) visually meaningful cipher image of one, two, three and four plain images, respectively.

Many researchers have introduced various technologies to design image encryption schemes, including DNA coding technology [1], [2], [3], [4], [5], chaos theory [6], [7], [8], [9], [10], [11], optical transformation technology [12], [13], [14], [15], cellular automata [16], [17], etc. These image encryption schemes often encrypt meaningful plain image into noise-like cipher image, as shown in Fig. 1(a). Since it is impossible to obtain information from the noise-like cipher image visually, and it is also difficult to obtain the key of the encryption system through ciphertext analysis, these encryption schemes achieve data security. However, information security includes data encryption and data hiding. The traditional encryption schemes encrypt plain images into noise-like cipher images only realize data encryption, but not realize data hiding. When the visually meaningless noise image appears on the insecure transmission channel, it is easy to attract the attention of attackers.

Steganography realizes information hiding by embedding private information into carrier media [18]. In steganography, an unintentional recipient is not suspicious of a host image that contains private information. There are two kinds of embedding methods, the spatial-domain embedding and the transform-domain embedding, in steganography [19], [20], [21], [22]. In order to enhancement the image quality, researchers have proposed many methods. Abdulla et al. proposed a two-step steganography scheme based on message size reduction and Fibonacci bit-plane mapping, which improves the stego image quality and secret message un-detectability without reducing the encryption capacity [23]. Sun proposed an embedding method, which is based on Canny edge detector and 2k correction [24]. Liao et al. proposed an adaptive steganography scheme for JPEG image, which maintains the correlation between adjacent coefficients between blocks by adjusting the cost values during embedding [25]. Tamer et al. proposed a high-capacity image hiding scheme to improve steganographic image quality by using multiscale Laplacian pyramid of the host image in the discrete wavelet transform domain [26]. Abdulla et al. further improved the embedding efficiency of steganography by using the similarity between the secret image and the host image [27]. Fawad et al. proposed a quality-enhanced and secure method of content-adaptive image steganography, and the scheme selectively embeds images with high texture content [28]. Neha et al. proposed an image steganography scheme based on Huffman coding and particle swarm optimization, which improved the performance and efficiency of information hiding [29].

For the purpose of hiding cipher images in the insecure transmission channel, Bao et al. proposed a new encryption framework, which realizes the synchronization of data encryption and data hiding by encrypting the plain image into a visually meaningful cipher image [30]. The scheme includes two phases: encryption phase and embedding phase. In the encryption phase, a plain image is encrypted into a secret image using traditional encryption algorithms. In the embedding phase, a host image (Fig. 1(b)) and the secret image are transformed into wavelet coefficients through discrete wavelet transform, then part of the host image wavelet coefficients are replaced with the secret image wavelet coefficients, and then the replaced wavelet coefficients are processed by inverse discrete wavelet transform to obtain a visually meaningful cipher image, which is similar to the host image. Since such a cipher image is visually indistinguishable from an ordinary image, when it is transmitted with other images on a public network, it is difficult to be considered as containing additional information even if it is intercepted. However, the introduction of the embedding process makes the size of the cipher image several times larger than that of the plain image. At the same time, the discrete wavelet transform used in the embedding process is irreversible, which makes the secret image lose some information during the embedding process, and further leads to the degradation of the decrypted image quality.

Compressive sensing (CS) theory provides a method for recovering the original signal from the measured signal at a lower sampling rate, which can realize the synchronization of data compression and encryption [31], [32], [33], [34], [35], [36]. To reduce the size of the visually meaningful cipher image, researchers have introduced CS into the encryption process. In these encryption schemes, firstly CS is used to compress and encrypt the plain image to obtain the secret image, and then the secret image is embedded into the host image to obtain the visually meaningful cipher image, in which the size of the cipher image is the same as that of the plain image [37], [38], [39]. In order to improve the quality of the decrypted image, Wang et al. used the completely reversible integer wavelet transform (IWT) instead of the irreversible discrete wavelet transform in the embedding phase [40]. Yang et al. proposed an improved visually meaningful encryption scheme for color images, Qi hyper-chaotic mapping is used in the scheme to enhance the security of the pre-encryption process [41]. Ye et al. proposed a visually meaningful image encryption scheme based on CS and information hiding, which combines wavelet transform and singular value decomposition to embed the secret image into the host image to form a visually meaningful cipher image [42]. Wang et al. proposed a novel triple-image encryption and hiding scheme based on a chaotic system, CS and transform domain embedding, which improved the transmission efficiency [43]. Isaac et al. proposed an image encryption scheme based on CS and adjustable gradient Hopfield neural network, in which the measurement matrix of the CS is designed using the sequences of the proposed chaotic system [44]. Chai et al. proposed a visually meaningful image compression and encryption scheme by combining CS and least significant bit embedding [45], and then they proposed a visually meaningful color image encryption scheme [46]. Besides, the traditional CS reconstruction algorithm takes a long time. In order to reduce the computational complexity, researchers have introduced block compressive sensing [47], 2D compressive sensing (2DCS) [48], [49], Bayesian compressive sensing [50] and parallel compressive sensing (PCS) [40], [51], [52] into the visually meaningful image encryption schemes. The visual security image encryption scheme includes the pre-encryption process (including CS, chaotic maps, and other operations) and the embedding process (including transformation, embedding and other operations). Table 1 compares some of the visually meaningful image encryption schemes.

**Table 1:** Comparison of different schemes.

Scheme	Pre-encryption process			Embedding process	
	CS	Chaotic maps	Other operation	Transform	Embedding
[37]	CS	Skew tent map	SHA 256, zigzag confusion	DWT	LH and HL
[40]	PCS	3-D cat map	Zigzag confusion	IWT	HL, LH and HH
[41]	No	Qi hyper-chaotic system	SVD	DWT, DCT	YCbCr color space
[42]	CS	Logistic-tent map	Random numbers insertion	2D-DWT	Singular values
[43]	CS	2D infinite collapse map	3D zigzag scrambling	3D-DCT	Lower right corner
[45]	CS	3-D Cat map	Zigzag confusion	DWT	Least significant bit
[47]	Block CS	Hyper-chaotic system	Counter mode	IWT	Singular values
[48]	2DCS	4-D hyperchaotic system	SHA 256 hash	IWT	Multi-embedding
[49]	2DCS	2D random scrambling	Grayscale transformation	IWT	LL, HL, LH and HH
[50]	Bayesian CS	2D fractional-order map	Arnold confusion	2D-DVT	DVT embedding
[51]	PCS	2D cat map	Adaptive threshold	2-layer SWT	Matrix encoding
[52]	PCS	4D memristive hyperchaos	Arnold scrambling	slant transform	ST-based embedding

### 1.2 Motivation

Although many research groups have made various improvements to the visual security encryption scheme based on CS, there are still some problems, such as: only one image is encrypted, and the embedding process is not flexible. In some cases, people hope that more plain images can be encrypted while the amount of ciphertext data remains the same. At the same time, they hope that the decrypted image and the plain image have a higher degree of similarity, but don't care about the similarity between the cipher image and the host image. Inspired by the above reasons, we propose a visually secure multi-image encryption scheme, which can flexibly realize the encryption of multiple plain images without increasing the amount of ciphertext data. As shown in Fig. 1(c)–(f), the scheme encrypts multiple plain images into a visually meaningful cipher image of the same size, and the larger the number of encrypted plain images, the lower the similarity between the visually meaningful cipher image and the host image.

### 1.3 Encryption scheme

In this paper, we propose a novel visually meaningful multi-image encryption scheme based on 2DCS. In the encryption process of one plain image, the plain image is pre-encrypted by randomly changing its pixel position and pixel value, then the pre-encrypted image is compressed through 2DCS to obtain a secret image. In the embedding process of one secret image, the host image and the secret image are transformed into wavelet coefficients through IWT, then the wavelet coefficients are decomposed into eight bit planes, and then the secret image wavelet coefficient is embedded by decomposing and replacing the lowest two bit planes (bit plane 1 and bit plane 2) of the host image wavelet coefficient to obtain the visually meaningful cipher image. The method of encrypting multiple plain images is analogous to this, and the extraction and reconstruction of multiple plain images do not affect each other. Digital simulation and comprehensive performance analysis show the effectiveness, data security and visual security of the multi-image encryption scheme.

The main contributions of our work lie in:

(1) Propose a visually secure multi-image encryption scheme, which can flexibly realize the encryption of multiple plain images without increasing the amount of ciphertext data.
(2) Introduce non-linear random gray value transformation process to improve the security of the scheme. This operation avoids the leakage of plaintext statistical information by changing the gray value of the image. At the same time, because this operation is a non-linear operation, the system can resist the chosen-plaintext attack. Furthermore, this operation maps the data to -128 to 128, which reduces the dynamic range of the sampled data acquired by 2DCS.
(3) According to specific needs, you can flexibly choose to improve the visual security of encrypted image or increase the number of plain images.

### 1.4 Organization

The rest of this paper is organized as follows. Section 2 gives the basic knowledge of CS. Section 3 describes the encryption scheme we proposed in detail. First, the embedding process of one secret image is given. Based on this, the encryption scheme of one plain image is described, and then it is extended to multiple plain images encryption scheme. Section 4 discusses the security of the encryption scheme in detail through numerical simulation and performance analysis. The last part summarizes the paper.

## 2 Preliminary work

### 2.1 1D compressive sensing

CS is a signal acquisition theory, which points out that when the signal satisfies the sparsity constraint, it can be recovered from the under-sampled measurement data [31], [32], [33], [34]. Generally speaking, for a one-dimensional signal *x* of length *N*, it can be expressed as Eq. (1)

(1) $$x=\sum_{i=1}^{N}\alpha_{i}\psi_{i}=\Psi \alpha$$

where $\alpha =\left[\alpha_{1},\ldots ,\alpha_{i},\ldots ,\alpha_{N}\right]$, and $\alpha_{i}=\langle x,\psi_{i}\rangle =\psi_{i}^{T}x$ is the projection coefficient of *x*. Ψ is an N×N sparse matrix. The incoherent measurement process can be expressed as Eq. (2) [31], [32], [33], [34]

(2) y=Φx=ΦΨα=Θα

where Φ is an M×N(M<N) measurement matrix, sensor matrix Θ=ΦΨ, and *y* is an M×1 under-sampled measurement data. The sparse signal *x* can be recovered from measurement data *y* by solving the optimization equation as Eq. (3)

(3) $$\min\;{\left\|\alpha \right\|}_{0},\;\text{subject to}\;y=\Phi x$$

where ${\left\|\alpha \right\|}_{0}$ is the number of nonzero components in *α* [31], [32], [33], [34].

### 2.2 2D compressive sensing

The compression sampling and reconstruction process of 2D image *X* is similar to that of one-dimensional signal *x*. The 2D image *X* with size N×N can be sparse represented as Eq. (4)

(4) $$\beta =\Psi X\Psi^{T}$$

where *β* is the coefficient matrix of *X* in Ψ domain, whose size is N×N.

The compression sampling process of the 2D image *X* requires two measurement matrices *A* and *B* of size M×N(M<N), and the process can be expressed as Eq. (5)

(5) $$Y=AXB^{T}$$

where the measurement data *Y* is an M×M matrix. Similarly, the 2D image *X* can be recovered from measurement data *Y* by solving the optimization equation as Eq. (6)

(6) $$\min{\left\|\beta \right\|}_{0}\text{ subject to}\;\;Y=A\beta B^{T}$$

There are many ways to solve the above equation, including 2D orthogonal matching pursuits algorithm (2DOMP) [53], 2D smoothed $L_{0}$ algorithm (2DS$L_{0}$) [54], 2D projected gradient algorithm (2DPG) [55], [56] etc.

## 3 The proposed encryption scheme

This paper proposes a visually meaningful multi-image encryption scheme based on 2DCS and bit-plane embedding, which can flexibly encrypt multiple plain images into a visually meaningful cipher image. The plain images, host images, secret images and cipher images processed in this paper are all 8-bit grayscale images. The principle of encrypting multiple plain images is similar to that of encrypting one plain image, except for the embedding process of the secret image. Below we will first describe one secret image embedding process, then describe the one plain image encryption scheme, and then on this basis, describe the encryption schemes for two, three, and four plain images. The scheme can also be extended to encrypt more plain images. It should be pointed out that this scheme has requirements on the sampling rate *η* and the maximum number of plain images *P*, which should be satisfied η×P≤1. In this paper, the sampling rate is set as 25%, so P≤4. Further reducing the sampling rate *η* can increase the maximum number of plain images *P*, but will reduce the quality of decrypted images.

### 3.1 One secret image embedding process

In this paper, the size of the host image and the plain image are both 256×256, and the sampling rate is set to 25%, so the size of the secret image obtained by 2DCS is 128×128.

**Figure 2:**
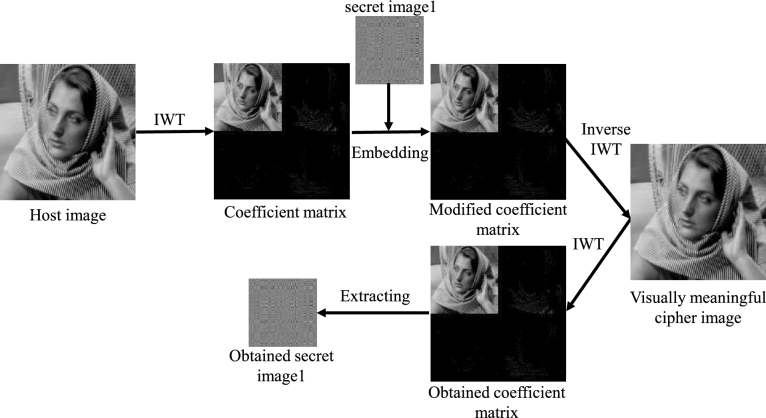
The embedding and extracting process of one secret image based on IWT.

**Figure 3:**
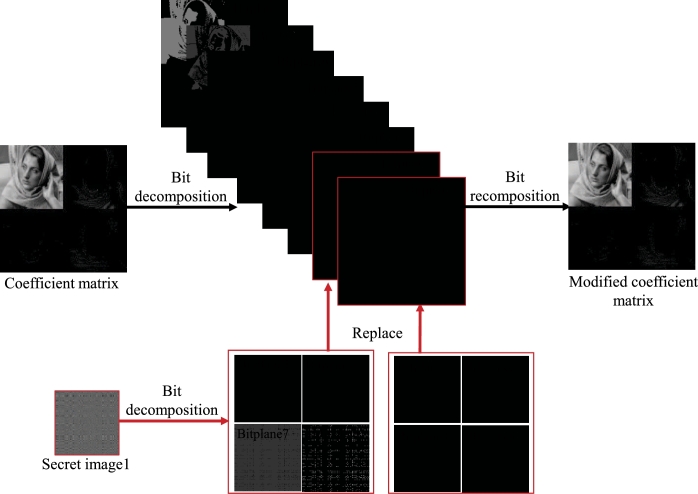
Embed one secret image into the coefficient matrix through bit-plane decomposition.

The embedding and extraction process of one secret image proposed in this paper is shown in Fig. 2, in which a fully reversible IWT is used, so that the secret image can be embedded and extracted from the host image without loss. For the embedding process, the measured data (*Y* in Eq. (5)) is quantized to obtain a secret image, which is an 8-bit grayscale image with the size of 128×128, then IWT operation is performed on the host image and quantization is performed to obtain the quantized wavelet coefficient, which is an 8-bit grayscale image with the size of 256×256, then the quantized wavelet coefficient are decomposed into eight bit planes, and then the secret image is embedded by decomposing and replacing the lowest two bit planes (bit plane 1 and bit plane 2) of the host image quantized wavelet coefficient to obtain a correction coefficient matrix, then it is transformed into spatial domain to obtain a visually meaningful cipher image. The detailed process of bit plane replacement is shown in Fig. 3. The quantized coefficient matrix (size is 256×256) and the secret image (size is 128×128) are decomposed bit by bit to obtain 8 bit planes, then the 8 bit planes of the secret image are synthesized into two matrices of size 256×256, and these two matrices are used to replace the coefficient matrix bit plane 1 and bit plane 2 of coefficient matrix. Finally, the 8 bit planes after completing the replacement process are synthesized into a modified coefficient matrix.

The extraction process of the secret image is the inverse process of the Fig. 3. First, perform bit decomposition on the modified coefficient matrix in Fig. 3 to obtain 8 bit planes, then extract bit plane 1 and bit plane 2, and divide each 256×256 bit plane into four 128×128 bit planes according to the method shown in Fig. 3, and finally 8 bit planes are combined to get a extracted secret image. Since the IWT and bit-plane decomposition used in this process are completely reversible, the embedded secret image and the extracted secret image are also exactly the same.

### 3.2 One plain image encryption scheme

**Figure 4:**
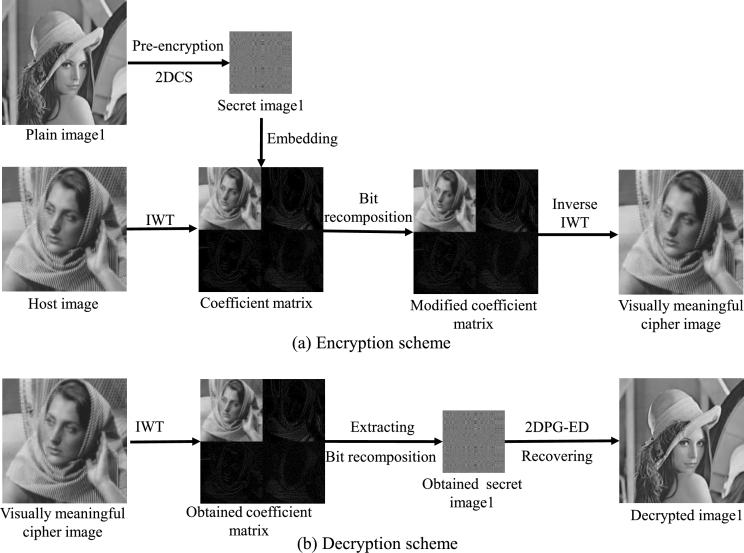
One plain image encryption and decryption scheme.

The one plain image encryption scheme is shown in Fig. 4(a), and the detailed steps are as follows.

Step 1: An index matrix *R* with size N×N is generated by randomperm function, which is used to scramble the plain image *X*. For example, if the index matrix R=[3,4;1,2], plain image $X=[x_{11},x_{12};x_{21},x_{22}]$, we scramble *X* to get $X^{\prime }=R(X)=[x_{21},x_{22};x_{11},x_{12}]$. This process is reversible and the index matrix *R* needs to be stored as a decryption key.

Step 2: The values of some elements in $X_{1}^{\prime }$ are randomly changed according to Eq. (7).

(7) $$X_{1}^{\prime \prime }(i,j)=\left\{ \begin{array}{c} X_{1}^{\prime }(i,j)-128,\;\;\;\;Q_{1}(i,j)=1\\ 127-X_{1}^{\prime }(i,j),\;\;\;\;Q_{1}(i,j)=0 \end{array}\right.$$

We call this nonlinear process the random gray value transformation. The size of binary random matrix $Q_{1}$ is the same as that of the plain image. $X_{1}^{\prime \prime }$ is a pre-encrypted integer matrix with element values from -128 to 128. This operation avoids the leakage of plaintext statistical information by changing the gray value of the image. At the same time, because this operation is a non-linear operation, the system can resist the chosen-plaintext attack. Furthermore, this operation maps the data to -128 to 128, which reduces the dynamic range of the sampled data acquired by 2DCS in the next step. Binary random matrix $Q_{1}$ needs to be stored as a decryption key.

Step 3: Using CS theory to perform 2D compression sampling on the pre-encrypted matrix $X_{1}^{\prime \prime }$:

(8) $$Y_{1}=A_{1}X_{1}^{\prime \prime }B_{1}^{T}$$

In Eq. (8), two measurement matrices $A_{1}$ and $B_{1}$ are of size M×N(M<N), $B_{1}^{T}$ is the transpose of matrix $B_{1}$, so $Y_{1}$ denotes 2D measurement value matrix with size M×M. The measurement matrices $A_{1}$ and $B_{1}$ need to be stored as decryption keys.

Step 4: Quantify the measured value matrix $Y_{1}$, then transform the matrix element values to integers in the range of 0 to 255 in accordance with Eq. (9)

(9) $$Y_{1q}=\left\lfloor \frac{255(Y_{1}-min\_Y_{1})}{max\_Y_{1}-min\_Y_{1}}\right\rfloor$$

Here $min\_Y_{1}$ denotes the minimum value and $max\_Y_{1}$ denotes the maximum value in $Y_{1}$, respectively. ⌊•⌋ is the rounding down function. The obtained $Y_{1q}$ is the secret image 1 in Fig. 4(a). Here $min\_Y_{1}$ and $max\_Y_{1}$ need to be stored as decryption keys.

Step 5: Decompose the secret image $Y_{1q}$ by bit to obtain 8 bit planes with size of 128×128, and synthesize them into two 256×256 bit planes according to the method shown in Fig. 3.

Step 6: IWT operation is performed on the host image and quantization (as shown in Eq. (9)) is performed to obtain a quantized wavelet coefficient, which is an 8-bit grayscale image with the size of 256×256, and then we decompose the quantized wavelet coefficient by bit to obtain 8 bit planes. The type of IWT needs to be stored as a decryption key.

Step 7: The two 256×256 bit planes obtained from the secret image in step 5 are used to replace the lowest two bit planes in the 8 bit planes obtained from the coefficient matrix decomposition in step 6, as shown in Fig. 3.

Step 8: Synthesize the 8 bit planes after the replacement process into a modified coefficient matrix, then perform the inverse quantization operation and inverse IWT to obtain a visually meaningful cipher image.

In fact, steps 1 to 4 correspond to the plain image encryption process, and steps 5 to 8 correspond to the secret image embedding process. The decryption process is the reverse process of the encryption process, which is shown in Fig. 4(b). First, the coefficient matrix is obtained by decomposing the visually meaningful cipher image using inverse IWT, then the coefficient matrix is bit decomposed and the lowest two bit planes are extracted to synthesize the secret image, then the obtained secret image is decrypted by a 2DCS recovery algorithm to obtain pre-encryption image, and then it is inversely scrambled to obtain a decrypted image.

It is difficult to detect the difference between the visually meaningful cipher image and the host image by naked eyes, and the quality of the decrypted image is very good. In order to quantitatively analyze the effect of the encryption and decryption scheme, we calculated the peak signal to noise ratio (PSNR) and correlation coefficient (CC) of the visually meaningful cipher image and the host image, as well as the plain image and the decrypted image, and they can be calculated by Eq. (10)

(10) $$PSNR=10\log\frac{255^{2}}{(1/n^{2})\sum_{i=1}^{n}\sum_{j=1}^{n}{\left[R_{1}(i,j)-X_{1}(i,j)\right]}^{2}}\;CC=\mathrm{cov}\;\left(R_{1},X_{1}\right){\left(\sigma_{R_{1}}\cdot \sigma_{X_{1}}\right)}^{-1}$$

where $R_{1}$ and $X_{1}$ stand for the decrypted image and its corresponding plain image, respectively. *σ* and *cov* represent the standard deviation and covariance of the two images, respectively.

The PSNRs and CCs of the cipher image and the decrypted image 1 are shown in Table 2. It should be pointed out that the PSNR and CC of the cipher image are calculated by comparing with the host image, while the PSNR and CC of the decrypted image are calculated by comparing with the corresponding plain image. The PSNR and CC of the decrypted image are 31.8340 dB and 0.9907 respectively, indicating that the similarity between the decrypted image and the plain image is very high. There are two reasons why the effect of decrypted image is so good: 1. Due to the use of IWT, the process of embedding and extracting secret image can be completed losslessly; 2. In the compression sampling process, the sampling rate is set higher, which makes the reconstruction effect of the decryption algorithm good. The PSNR of the visually meaningful cipher image are 28.4667 dB, and the CC is 0.9820. This result shows that the cipher image and the host image are very close, and it is difficult to see the difference between the two from the appearance. The reason for the high similarity between the cipher image and the host image is that during the embedding process, only the lowest two bit planes of the host image coefficient matrix are replaced, and the other 6 high bit planes remain unchanged, as shown in Fig. 3.

**Table 2:** The PSNRs and CCs of the cipher image and the decrypted image 1.

	cipher image	decrypted image 1
PSNR	28.4667	31.8340
CC	0.9820	0.9907

### 3.3 Two plain images encryption scheme

**Figure 5:**
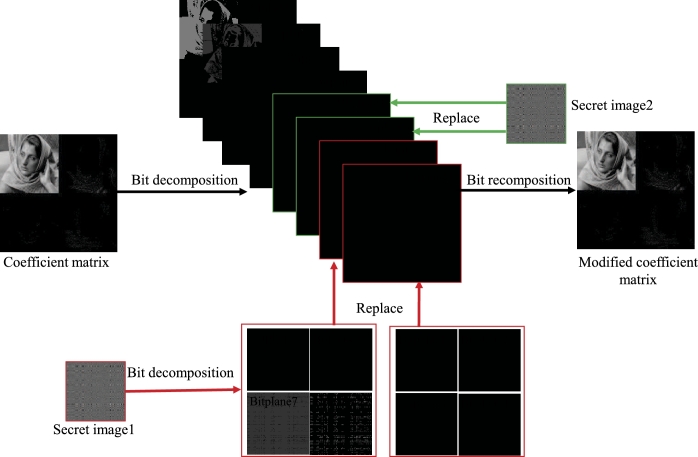
Embed two secret images into the coefficient matrix through bit-plane decomposition.

The encryption scheme for two plain images is roughly the same as the encryption scheme for one plain image, except for the embedding process of the two secret images. It is divided into two plain images encryption process and two secret images embedding process. In the two plain image encryption process, following the steps 1 to 4 in section 3.1, we encrypt the two plain images ($X_{1}$ and $X_{2}$) respectively to obtain the two secret images ($Y_{1q}$ and $Y_{2q}$). The keys used to encrypt the two plain images are different. In the two secret images embedding process, following the steps 5 to 8 in section 3.1, two secret images are embedded in coefficient matrix bit planes at different positions. Secret image 1 replaces coefficient matrix bit plane 1 and bit plane 2, and secret image 2 replaces coefficient matrix bit plane 3 and bit plane 4, as shown in Fig. 5. Finally, perform inverse IWT transformation on the modified coefficient matrix to obtain a cipher image.

**Figure 6:**
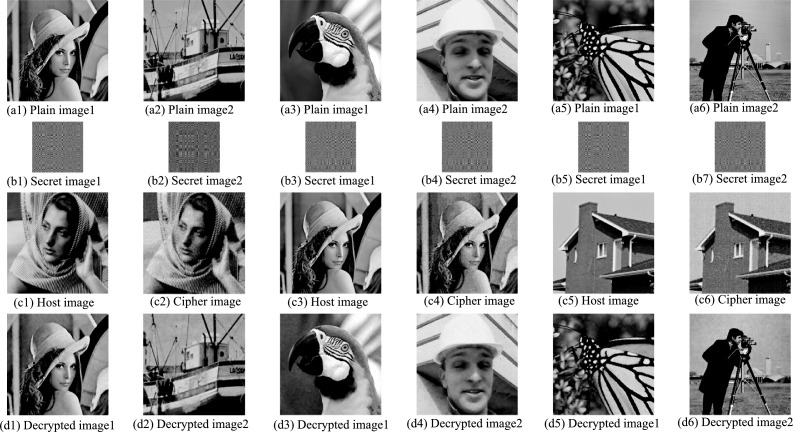
Three groups of encryption and decryption results of the two plain image encryption scheme: the first and second columns are the first group; The third and fourth column are the second group; The fifth and sixth columns are the third group.

Three groups of the encryption and decryption results are shown in Fig. 6: The first row is 6 plain images (Fig. 6(a1)–(a6)), the second row is the 6 secret images (Fig. 6(b1)–(b6)) corresponding to the plain images in the first row, the third row is the host images (Fig. 6(c1), Fig. 6(c3) and Fig. 6(c5)) and the cipher images (Fig. 6(c2), Fig. 6(c4) and Fig. 6(c6)), and the last row is the 6 decrypted images (Fig. 6(d1)–(d6)) obtained by decrypting the corresponding cipher images. The PSNRs and CCs of the cipher image and the two decrypted images are shown in Table 3. It can be seen that the cipher image is visually similar to the corresponding host image, and its average PSNR=26.0975dB and CC=0.9656. However, compared with the cipher image of one plain image (in Fig. 4(a)), whose PSNR=28.4667 dB and CC=0.9820, the clarity of the cipher image of the two plain images (Fig. 6(c2)) is decreased. The reason for the decrease in the similarity between the cipher image and the host image is the embedding process. In the embedding process of the two plain image encryption scheme, four bit planes of the host image coefficient matrix are replaced. In the embedding process of one plain image encryption scheme, only two bit planes of the coefficient matrix are replaced. Therefore, as the number of encrypted plain images increases, the PSNR and CC values of cipher image decrease.

**Table 3:** The PSNRs and CCs of the cipher image and the two decrypted images.

Group	Plain images	Host images	Cipher images		Decrypted images	
			PSNR(dB)	CC	PSNR(dB)	CC
First Group	Lena	Barbara	25.2063	0.9621	31.8340	0.9907
	Boats				31.0316	0.9896
Second Group	Parrots	Lena	26.1015	0.9652	33.6709	0.9959
	Foreman				35.8861	0.9971
Third Group	Monarch	House	26.9848	0.9694	30.5565	0.9901
	Cameraman				30.0962	0.9918
Average value			26.0975	0.9656	32.1792	0.9925

It can be seen that the PSNRs and CCs of the decrypted images are very high, indicating that the quality of the decrypted images is good. The calculated PSNR of the decrypted image 1 is 31.8340 dB, and the CC is 0.9907, and this result is the same as the result in the single image encryption scheme in section 3.1. This is because the embedding process of the two secret images is independent of each other, that is, the process of embedding the secret image 2 into the host image does not affect the embedding and extraction of the secret image 1 at all, so the decrypted image 1 is completely consistent with the result of the one plain image encryption scheme.

### 3.4 Three plain images encryption scheme

**Figure 7:**
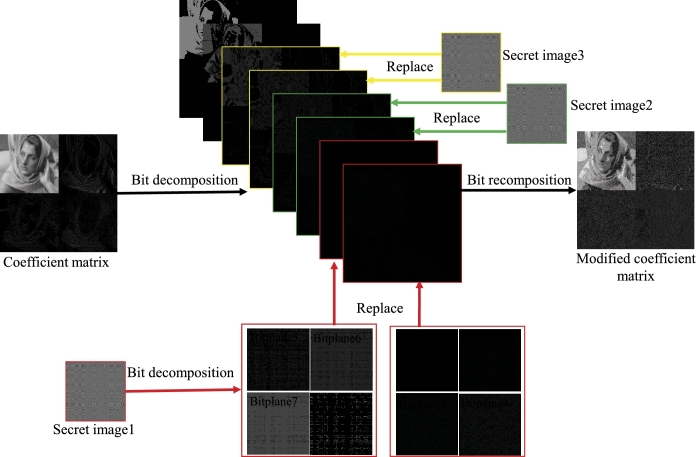
Embed three secret images into the coefficient matrix through bit-plane decomposition.

Similarly, the encryption scheme for three plain images is divided into encryption process and embedding process. Three plain images are respectively encrypted during the encryption process, and the embedding process embeds the three secret images into the host image coefficient matrix (as shown in Fig. 7). In the encryption process, following the steps 1 to 4 in section 3.1, we encrypt the plain image 1 ($X_{1}$), the plain image 2 ($X_{2}$) and the plain image 3 ($X_{3}$) respectively to obtain the secret image 1 ($Y_{1q}$), the secret image 2 ($Y_{2q}$) and the secret image 3 ($Y_{3q}$). To ensure the security of the system, the keys used to encrypt the three plain images are different. The embedding process involves the embedding of three secret images. The secret image 1 and the secret image 2 are embedded in the coefficient matrix, following the embedding process in section 3.2. Secret image 3 is embedded in coefficient matrix bit planes at different positions. Secret image 1 replaces coefficient matrix bit plane 1 and bit plane 2, and secret image 2 replaces coefficient matrix bit plane 3 and bit plane 4, and secret image 3 replaces coefficient matrix bit plane 5 and bit plane 6, as shown in Fig. 7. Finally, perform inverse IWT transformation on the modified coefficient matrix to obtain a cipher image.

**Figure 8:**
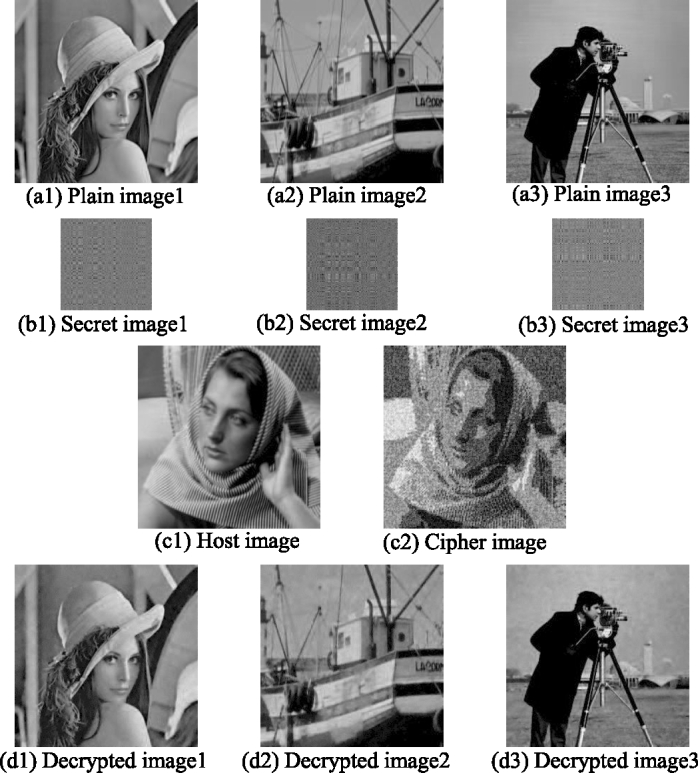
Three plain images encryption and decryption results.

The encryption and decryption results are shown in Fig. 8: The first row is 3 plain images (Fig. 8(a1)–(a3)), the second row is the 3 secret images (Fig. 8(b1)–(b3)) corresponding to the plain images in the first row, the third row is the host image (Fig. 8(c1)) and the cipher image (Fig. 8(c2)), and the last row is the 3 decrypted images (Fig. 8(d1)–(d3)) obtained by decrypting the cipher image. The PSNRs and CCs of the cipher image and the three decrypted images are shown in Table 4. It can be seen that the cipher image is visually similar to the host image, and its PSNR=16.1648 dB and CC=0.7717. However, compared with the cipher image of two plain images (Fig. 6(c2)), whose PSNR=25.2063 dB and CC=0.9621, the clarity of the cipher image of three plain images (Fig. 8(c2)) is decreased. The reason for the decrease in the similarity between the cipher image and the host image is the embedding process. In the embedding process of the two plain image encryption scheme, four bit planes of the host image coefficient matrix are replaced. In the embedding process of three plain images encryption scheme, six bit planes of the coefficient matrix are replaced. Therefore, as the number of encrypted plain images increases, the number of replaced bit planes in the coefficient matrix increases, and the similarity between the encrypted image and the host image decreases.

**Table 4:** The PSNRs and CCs of the cipher image and the three decrypted images.

	cipher image	decrypted image 1	decrypted image 2	decrypted image 3
PSNR	16.1648	31.8340	31.0316	29.9095
CC	0.7717	0.9907	0.9896	0.9915

### 3.5 Four plain images encryption scheme

**Figure 9:**
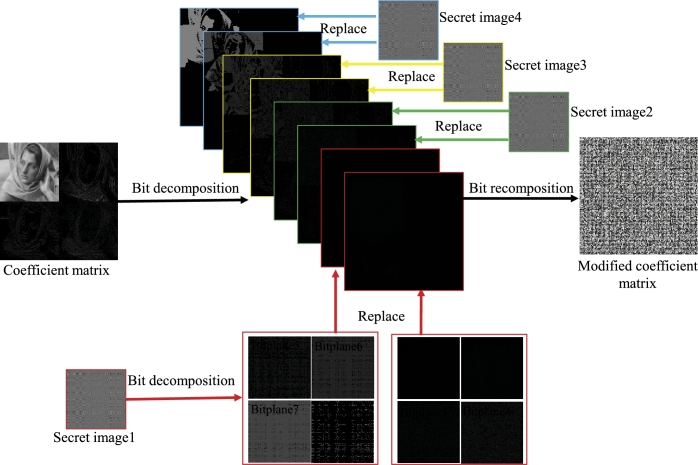
Embed four secret images into the coefficient matrix through bit-plane decomposition.

**Figure 10:**
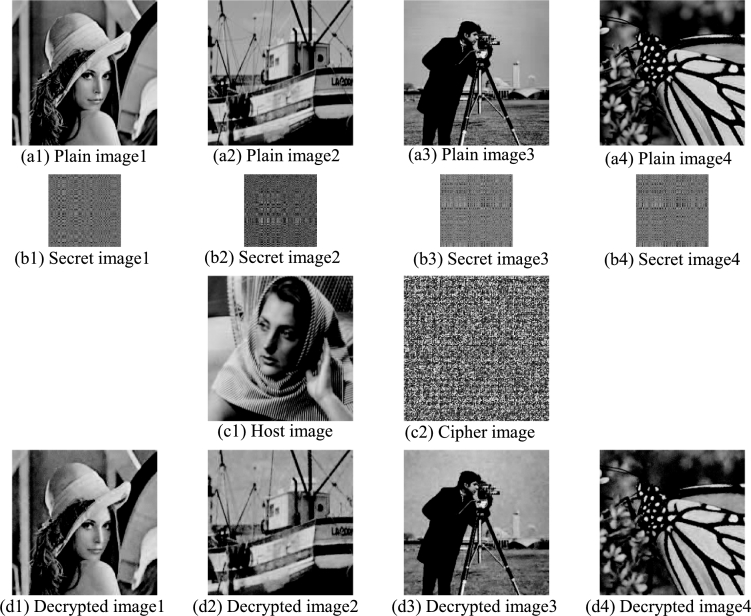
Four plain images encryption and decryption results.

Similarly, if four plain images are respectively encrypted during the encryption process, and the embedding process embeds the four secret images into the host image coefficient matrix (as shown in Fig. 9), an encryption scheme for four plain images is obtained. The encryption and decryption results are shown in Fig. 10: The first row is 4 plain images (Fig. 10(a1)–(a4)), the second row is the 4 secret images (Fig. 10(b1)–(b4)) corresponding to the plain images in the first row, the third row is the host image (Fig. 10(c1)) and the cipher image (Fig. 10(c2)), and the last row is the 4 decrypted images (Fig. 10(d1)–(d4)) obtained by decrypting the cipher image. The PSNRs and CCs of the cipher image and the four decrypted images are shown in Table 5. It can be found that the cipher image and the host image are not visually related at this time, and its PSNR=7.5401 dB and CC=0.0077. This is because the four secret images completely replaced the eight bit planes of the host image coefficient matrix during the embedding process, and the obtained correction coefficient matrix has nothing to do with the host image coefficient matrix, as shown in Fig. 9. So, the cipher image obtained by transforming the correction coefficient matrix into the spatial domain has nothing to do with the host image.

**Table 5:** The PSNRs and CCs of the cipher image and the four decrypted images.

	cipher image	decrypted image 1	decrypted image 2	decrypted image 3	decrypted image 4
PSNR	7.5401	31.8340	31.0316	29.9095	29.8356
CC	0.0077	0.9907	0.9896	0.9915	0.9884

It can be seen from Fig. 10 that the quality of the four decrypted images is very good, and the PSNRs and CCs are very high. In the four plain images encryption scheme, the cipher image has nothing to do with the host image, because the eight bit planes of the host image coefficient matrix are completely replaced by four secret images. At this time, no visually meaningful cipher image is obtained, but the number of encrypted images reaches the maximum.

The multi-image encryption scheme can flexibly encrypt multiple plain images of equal size in a cipher image without reducing the decryption quality of the decrypted image at all. As the number of encrypted images increases, the quality of decrypted images will not decrease. The more encrypted images, the lower the similarity between the encrypted image and the host image.

## 4 Security analysis and discussion

Next, we will use Matlab R2019b to simulate the proposed encryption scheme on the win10 platform with 3.0 GHz CPU and 32 GB RAM. The feasibility and effectiveness of the scheme are verified through histogram analysis, correlation coefficient analysis, information entropy analysis and resistance to common attacks on cipher image.

### 4.1 Histogram analysis

**Figure 11:**
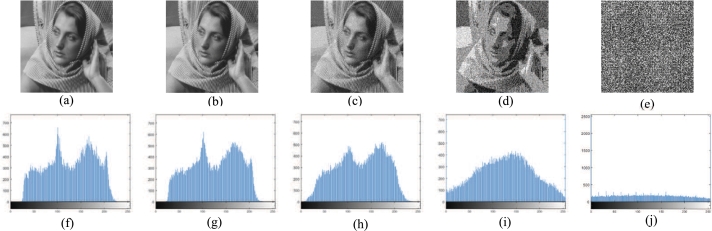
Histogram analysis: (a) host image; (b)–(e) cipher images corresponding to one, two, three and four plain images, (f)–(j) corresponding histograms.

The pixel value distribution information of the image can be obtained from the histogram. The traditional encryption algorithm encrypts the plain image into a noise-like cipher image, and the pixel value distribution of the cipher image is uniform and flat. However, such cipher image is more likely to attract the attention of attackers and suffer more cryptanalysis. Fig. 11 shows the host image and cipher images corresponding to one, two, three and four plain images proposed in section 3. Fig. 11(a) is the host image, Fig. 11(b)–(e) are the visually meaningful cipher images corresponding to one, two, three and four plain images, respectively. It can be found that the cipher images Fig. 11(b)–(d) are visually similar to the host image, and Fig. 11(e) has no visual correlation with the host image. Fig. 11(f)–(j) are the histograms corresponding to Fig. 11(a)–(e). Fig. 11(g) (the histogram of cipher image of one plain image) and Fig. 11(f) (the histogram of host image) is very similar and it is difficult to find the difference. As the number of encrypted plain images increases, the difference in histograms increases. The outline of the cipher images obtained by encrypting two and three plain images is still visually similar to that of the host image, but their histograms are quite different, as shown in Fig. 11(h) and Fig. 11(i). Since Fig. 11(e) has no visual correlation with the host image, its corresponding histogram (Fig. 11(j)) has nothing to do with that of the host image (Fig. 11(f)). There is a trade-off between the number of encrypted plain images and the strength of visual security, depending on the specific situation.

### 4.2 Correlation analysis of adjacent pixels

In order to quantitatively analyze the correlation, we select 3000 pairs of adjacent pixels $\left(x_{i},y_{i}\right)$ randomly from the host image and the cipher images corresponding to one, two, three and four plain images (Fig. 11(a)–(e)), and calculate the correlation coefficients (horizontally, vertically and diagonally adjacent) according to Eq. (11)

(11) $$Cor=\frac{\sum_{i=1}^{N}\left(x_{i}-E(x)\right)\left(y_{i}-E(y)\right)}{\sqrt{\sum_{i=1}^{N}{\left(x_{i}-E(x)\right)}^{2}\times \sum_{i=1}^{N}{\left(y_{i}-E(y)\right)}^{2}}}$$

where $E(x)=\frac{1}{N}\sum_{i=1}^{N}x_{i}$ and $E(y)=\frac{1}{N}\sum_{i=1}^{N}y_{i}$. $x_{i}$ and $y_{i}$ are the values of the selected adjacent pixels, and *N* is the total number of pixel pairs. Correlation coefficients of horizontally, vertically and diagonally directions are shown in Table 6, respectively.

**Table 6:** Correlation coefficients of adjacent pixels.

Image	Horizontal	Vertical	Diagonal
Fig. 11(a)	0.9095	0.9684	0.8400
Fig. 11(b)	0.8894	0.9645	0.8490
Fig. 11(c)	0.8842	0.9383	0.8219
Fig. 11(d)	0.6440	0.7123	0.4821
Fig. 11(e)	0.0074	0.0326	-0.3189

**Figure 12:**
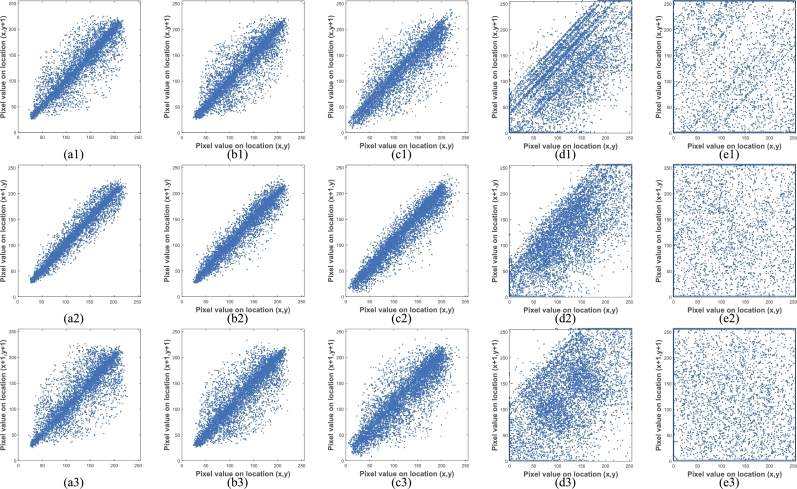
Correlation coefficient analysis: from the 1st column to the 5th column, the horizontal, vertical and diagonal correlation diagrams of Fig. 11(a)–11(e) are listed respectively.

Fig. 12 reflects the horizontal, vertical and diagonal correlation of adjacent pixels in Fig. 11(a)–(e): The first column (Fig. 12(a1)–(a3)) is the correlation of adjacent pixels in Fig. 11(a), the second column (Fig. 12(b1)–(b3)) is the correlation of adjacent pixels in Fig. 11(b), the third column (Fig. 12(c1)–(c3)) is the correlation of adjacent pixels in Fig. 11(c), the fourth column (Fig. 12(d1)–(d3)) is the correlation of adjacent pixels in Fig. 11(d), the fifth column (Fig. 12(e1)–(e3)) is the correlation of adjacent pixels in Fig. 11(e). It can be seen that the correlation distribution between the host image and the cipher image of one and two plain images is not much different, but it is very different from the cipher image of the three and four plain images. From Table 6 we can find that when encrypting one or two plain images, the correlation coefficients of the cipher images are very close to that of the host image. When encrypting three plain images, the correlation coefficients in each direction of the obtained cipher image (Fig. 11(d)) and that of the host image are quite different. When encrypting four plain images, there is no relationship between correlation coefficients in all directions of the cipher image (Fig. 11(e)) and that of the host image. In addition, even if the number of encrypted images is large, it is difficult to distinguish whether the image contains noise or other meaningful information. Therefore, the multi-image encryption scheme can achieve the visual security of cipher images very well.

### 4.3 Visual security and encryption capacity

We also studied the PSNR and CC of the cipher images (i.e. (b)–(e) in Fig. 11) obtained by embedding one, two, three and four plain images into the host image, and the results are shown in Table 7. It can be seen that when only one plain image is embedded into the host image, the PSNR and CC of the cipher image obtained are 28.4399 dB and 0.9818, indicating that the cipher image has high similarity with the host image and strong visual security. As the number of embedded images increases, the similarity between cipher image and host image decreases and its visual security decreases. When there are four plain images embedded in the host image, the PSNR and CC of the cipher image obtained are 7.5401 dB and 0.0077, indicating that the cipher image is completely irrelevant to the host image and has no visual security. Users can compromise between visual security and encryption capacity based on their actual needs.

**Table 7:** The PSNRs and CCs of the four cipher images.

	Fig. 11(b)	Fig. 11(c)	Fig. 11(d)	Fig. 11(e)
PSNR	28.4399	25.1506	16.1648	7.5401
CC	0.9818	0.9616	0.7717	0.0077

### 4.4 Information entropy analysis

Information entropy can reflect the randomness and unpredictability of information sources. The definition of information entropy H(x) is as Eq. (12)

(12) $$H(x)=\sum_{i=0}^{2^{n-1}}p\left(x_{i}\right)\log\frac{1}{p\left(x_{i}\right)}$$

where *n* is the number of bits representing the symbol $x_{i}$, and $p(x_{i})$ is the probability of $x_{i}$. When the probability of each pixel value is equal, the information entropy H(x) has a maximum value. For an 8-bit grayscale image, the maximum value is 8.

We calculated the information entropy of the four plain images (the first row), secret images (the second row) and decrypted images (the fourth row) in Fig. 10, and the results are shown in Table 8. It can be seen from Fig. 10 that secret images have certain texture features and their information entropy is low (about 6.2), indicating that the 2DCS-based pre-encryption algorithm fails to encrypt the plain image into a stationary white noise image. Since secret images are not transmitted directly over insecure channels, this does not affect visual security. It can be seen from Table 8 that the difference between the information entropy of the decrypted image and that of the plain image is very small, indicating that even if the multi-image encryption scheme encrypts and decrypts four images, its encryption and decryption effect is still very good.

**Table 8:** The information entropy of four plain images, secret images and decrypted images in Fig. 10.

Entropy	Plain image	Secret image	Decrypted image
Lena	7.4442	6.1742	7.4155
Boats	7.1456	6.2162	7.1518
Cameraman	7.0097	6.3608	7.1568
Monarch	7.4716	6.1730	7.4504

We also calculated the information entropy of the host image and the corresponding cipher image of one, two, three, and four plain images in Fig. 11, and the results are shown in Table 9. It can be seen that the difference between the information entropy of Fig. 11(a), (b) and (c) is very small, indicating that when the number of encrypted images is small, it is impossible to distinguish between the cipher image and the host image by calculating the information entropy. The information entropy of the cipher image (Fig. 11(d)) obtained by encrypting three plain images is 7.7944, which is significantly higher than that of the host image (7.5252). It is worth noting that the information entropy of the cipher image (Fig. 11(e)) obtained by encrypting the four plain images is only 6.4813. The reason for the lower value is that the minimum value 0 and maximum value 255 in Fig. 11(e) appear frequently, and the pixel value distribution is uneven, as shown in Fig. 11(j). It cannot achieve visual security, but still cannot see the information related to the plain images from the cipher image, so its data security can be achieved, and its advantage is that the number of encrypted images is the largest.

**Table 9:** The information entropy of host image and four cipher images in Fig. 11.

Image	Entropy
Fig. 11(a)	7.5252
Fig. 11(b)	7.5129
Fig. 11(c)	7.5825
Fig. 11(d)	7.7944
Fig. 11(e)	6.4813

### 4.5 Chosen-plaintext attack

**Figure 13:**
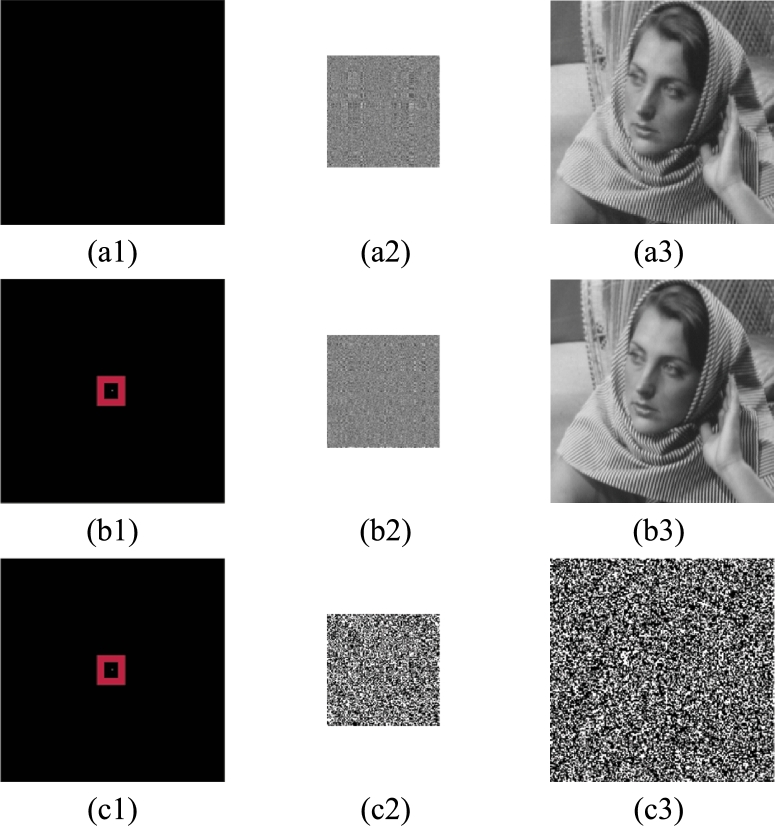
Chosen-plaintext attack and differential attack of one plain image encryption scheme. (a1) “all-zero” image, (a2) secret image of (a1), (a3) cipher image of (a1); (b1) “delta” image, (b2) secret image of (b1), (b3) cipher image of (b1); (*ci*)=255 × ((*bi*)−(*ai*)).

Chosen-plaintext attack means that the attacker can construct any cipher image corresponding to the plain image. For encryption schemes with scrambling operation, if all the pixel values of an image are equal, the scrambling operation can be invalidated. Therefore, we often choose to use an all-zero image (all black, as shown in Fig. 13(a1)) to attack the scrambling operation. The multiple plain image encryption scheme is similar to the one plain image encryption scheme. Below we will take the one plain image encryption scheme as an example, choose an all-zero image to try to crack the encryption scheme, and analyze the resistance of the scheme to chosen-plaintext attack.

For an all-zero image, the scrambling operation in the encryption process (the first step in Section 3.1) is invalid. In step 2, the all-zero image is non-linearly converted into a binary matrix with only element values 127 and -128 through Eq. (7), then 2DCS is used to compress and encrypt the binary matrix to obtain a secret image, as shown in Fig. 13(a2). Finally, the embedding of the secret image is completed by bit-plane decomposition and replacement, and the cipher image is obtained, as shown in Fig. 13 (a3), and its appearance is not different from the host image. The simulation results show that the plain image and encryption keys cannot be obtained through the chosen-plaintext attack.

### 4.6 Differential attack

A good encryption scheme must be able to resist differential attack, that is to say, it must be very sensitive to small differences in plain images. Sensitivity can be quantitatively evaluated by Number of Pixels Change Rate (NPCR) and Unified Average Changing Intensity (UACI), which are defined as Eq. (13)

(13) $$\begin{array}{c} \text{NPCR}=\frac{1}{W\times H}\sum_{i=1}^{W}\sum_{j=1}^{H}D\left(i,j\right)\times 100\text{\%}\\ \text{UACI}=\frac{1}{W\times H}\sum_{i=1}^{W}\sum_{j=1}^{H}\frac{\left|C_{1}\left(i,j\right)-\left|C_{2}\left(i,j\right)\right|\right|}{255}\times 100\text{\%} \end{array}$$

where $D(i,j)=\left\{ \begin{array}{cc} 0\; & \text{ if }c_{1}(i,j)=c_{2}(i,j)\\ 1\; & \text{ if }c_{1}(i,j)\neq c_{2}(i,j). \end{array}\right.$ W and H represent the width and height of the cipher image respectively, and $c_{1}(i,j)$ and $c_{2}(i,j)$ respectively represent the pixel value of cipher image 1 and cipher image 2 at (i,j).

By changing a pixel value of the plain image “zero” (Fig. 13(a1)), the modified plain image “delta” (Fig. 13(b1)) is obtained, then we encrypt it using the same method and same key to obtain a secret image (Fig. 13(b2)), and then embed it in the host image (Fig. 11(a)) to obtain a cipher image (Fig. 13(b3)). We calculate the NPCR and UACI of the two cipher images (Fig. 13 (a3) and (b3)) according to Eq. (13), and get NPCR=81.87%, UACI=0.65%. This result shows that when only one pixel value in the plain image changes, the pixel value in the obtained cipher image will change 81.87%, but the average change intensity of each pixel value is very low, only 0.65%. Therefore, the appearance of the cipher image remains almost unchanged.

For a more intuitive explanation, Fig. 13(ci) calculates the difference between (ai) and (bi) magnified 255 times. Obviously, when the difference between two plain images is only one pixel value, the difference value of the corresponding two secret images changes non-linearly, which in turn causes the difference value of the two cipher images to also change non-linearly, as shown in Fig. 13(c2) and (c3). This is because the Eq. (7) (step 2 in section 3.1) is a nonlinear equation during the encryption process. Therefore, the scheme can resist differential attack.

## 5 Conclusion

A visually meaningful multi-image encryption scheme based on 2DCS is proposed, which can flexibly complete the compression and encryption of multiple images without increasing the amount of ciphertext data. The scheme is divided into encryption process and embedding process. In the encryption process of one plain image, the plain image is pre-encrypted first, and then the pre-encrypted integer matrix is compressed by 2DCS to obtain a secret image. In the process of embedding one secret image, IWT, bit-plane decomposition and replacement are used to embed the secret image into the host image to obtain the cipher image. For the encryption of multiple images, only corresponding secret image needs to be embedded in different bit planes of the coefficient matrix. Compared with the traditional visually meaningful image encryption scheme, this scheme is more flexible and has a larger number of encrypted images. The scheme introduces a multi-secret image embedding mechanism, and realizes the encryption of multiple images without increasing the amount of ciphertext data. The simulation results and comprehensive analysis show that the scheme can resist histogram analysis attack, correlation analysis attack, information entropy analysis attack, chosen-plaintext attack and differential attack, and the greater the number of plain images, the lower the similarity between the cipher image and the host image, but it does not affect the quality of the decrypted image.

## Funding statement

Xin Zhou was supported by 10.13039/501100001809National Natural Science Foundation of China [61475104; 61177009]. Yanzhi Bai was supported by 10.13039/501100001809National Natural Science Foundation of China [52004159]. Chao Han were supported by 10.13039/501100001809National Natural Science Foundation of China [U22A2079], 10.13039/501100014762Anhui Polytechnic University [DTESD 2020A06]. Dongming Huo was supported by 10.13039/501100010814Anhui Provincial Department of Education [KJ2020 A0346], 10.13039/501100014762Anhui Polytechnic University [Xjky2022047; 2019 YQQ007]. Lisheng Wei was supported by 10.13039/501100010814Anhui Provincial Department of Education [KJ2020 ZD39], 10.13039/501100014762Anhui Polytechnic University [DTESD 2020A02].

## CRediT authorship contribution statement

Dongming Huo: Conceived and designed the experiments; Performed the experiments; Wrote the paper. Zhilong Zhu; Yanzhi Bai: Analyzed and interpreted the data. Xin Zhou: Conceived and designed the experiments. Lisheng Wei: Performed the experiments. Xing Bai: Contributed reagents, materials, analysis tools or data. Chao Han: Conceived and designed the experiments; Analyzed and interpreted the data.

## Declaration of Competing Interest

The authors declare no competing interests.

## Data Availability

Data will be made available on request.
